# The evaluation of the factors that cause aggregation during recombinant expression in *E. coli *is simplified by the employment of an aggregation-sensitive reporter

**DOI:** 10.1186/1475-2859-5-28

**Published:** 2006-09-01

**Authors:** Tina Schultz, Lucia Martinez, Ario de Marco

**Affiliations:** 1 EMBL Scientific Core Facilities, Meyerhofstr. 1, D-69117, Heidelberg, Germany; 2 IFOM-IEO Campus, Biochemistry Unit, via Adamello 16, I-20139, Milano, Italy

## Abstract

**Background:**

The yields of soluble recombinant proteins expressed in bacteria are often low due to the tendency of the heterologous proteins to form aggregates. Therefore, aggregation reporters have been envisaged to simplify the comparison among different expression conditions and to speed up the identification of suitable protocols that improve the solubility. The probe we used is composed by an *Ibp*AB promoter specifically activated by protein aggregates fused to a sequence coding the β-galactosidase, the activity of which becomes, therefore, indicative of the aggregation degree.

**Results:**

The collected data show that the probe is reliable in terms of reproducibility inside a range of experimental conditions and faster and more sensitive than the analysis methods based on SDS-PAGE and successive western blot.

The β-galactosidase probe was useful to identify which parameters could influence the aggregation of the model proteins and to set up an optimized expression protocol. The effect of growth temperature, induction modality, co-expression with molecular chaperones and addition of osmolytes on the accumulation of aggregates were evaluated following the β-galactosidase activity. Interestingly, a significant correlation was observed between estimated decreased aggregation and higher yields of soluble protein.

We also compared a set of expression vectors with various regulative features and found that the single characteristics, like promoter, copy number or polymerase, were not relevant for controlling the recombinant protein aggregation whilst the crucial factor resulted being the total expression rate of the system.

**Conclusion:**

The aggregation reporter used in our experiments represents a useful tool to evaluate the different factors that can be modulated to optimize a recombinant expression protocol. Furthermore, the rapid estimation of the aggregation degree enables to discriminate this from other causes responsible for scarce recombinant yields.

## Background

Aggregation is a disappointing, but frequent and quantitative relevant, shortcoming during recombinant expression in *E. coli*. The reasons for which even proteins with apparently simple structure fail to fold correctly remain unknown. A constantly increasing number of expedients have been proposed to improve the solubility of single proteins. However, their successful application to any further candidate remains unpredictable. Considering such reasons, and the always easier accessibility to automation that can speed up several growth and purification steps, systematic approaches have been proposed. They are based on small scale combinations of different variables like growth conditions, lysis buffers, type of constructs, host strains, fusion tags, co-expression of molecular chaperones and addition of osmolytes to identify promising solutions to be verified in large scale purification [1-8]. The resulting numerous samples can be screened using protocols based on SDS-PAGE or dot-blot to discriminate between promising and negative results [8-10].

Molecular probes capable to indicate the solubility of the target protein independently of a separation step can also be used. GFP has been fused to the C-term of the target proteins and the fluorescence of the resulting fusion protein considered indicative of its correct folding [11]. Similarly, fusions with β-galactosidase [12], chloramphenicol acetyltransferase [13] or the structural complementation between the C-term fused α-fragment of the β-galactosidase with the ω-fragment [14] have been described. However, the systems relying on GFP have at least two strong limitations, namely the long lag-phase (95 min) necessary to the chromophore to form and the persistence of the fluorescence even after protein aggregation [15,16]. In this context, the strategies exploiting the measurement of enzymatic activities seem, therefore, more reliable.

**Figure 1:**
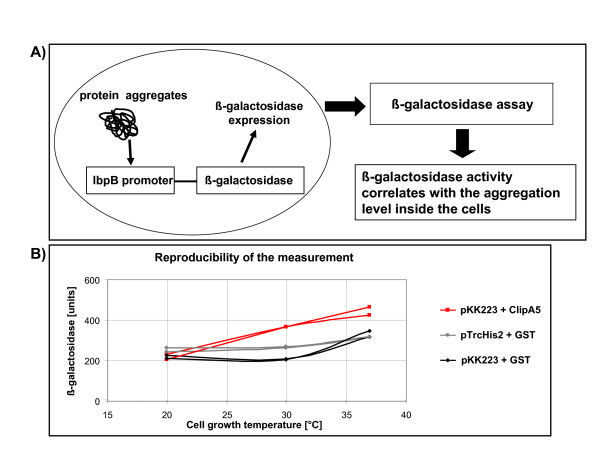
Mechanism of function and reliability of the protein aggregate-dependent probe. A) The native promoter of the β-galactosidase enzyme has been substituted by the aggregate-inducible *IbpB *promoter. As the consequence of protein aggregate crowding, the β-galactosidase accumulates and its activity serves as an indirect measurement of the aggregate amount in the cell [17]. B) Double independent measurements of the β-galactosidase activity at 3 different temperatures using 3 different constructs.

All these methods involve the production of fusion constructs that can modify the intrinsic solubility of the target protein. In an alternative approach a probe is used, the expression of which depends specifically on the presence of the aggregates, whilst it does not interact directly with the target protein. Lesley and co-workers [17] first identified genes activated by protein misfolding and then used their promoters fused to β-galactosidase to create reporters of the aggregation degree (Fig. 1A). This approach allowed the correct identification of soluble constructs in more than 80% of the more than 200 examined cases. However, its use was limited to yes/no screenings and no effort was dedicated to use the probe for collecting information aimed at improving the expression system [17].

Now we used one of these probes, the *Ibp*AB-promoter β-galactosidase fusion, to further test and exploit the potentialities of the method. The first set of data allowed us to determine the probe sensitivity and showed that it was apt to appreciate small variations of aggregate concentrations that are below the detection limit of the western blot. Next, the probe was used for comparing the level of aggregates accumulated when the recombinant protein was expressed from vectors with different translational regulation. It was also possible to select growth conditions that limit the aggregation of the recombinant protein during expression in *E. coli *and, finally, the factors influencing the probe reliability and which could generate inconsistent data are discussed.

## Results and discussion

### Reliability of the β-galactosidase probe

In a preliminary experiment thioredoxin (12 kD), NusA (54 kD), GST (27 kD and the fusion GST-GFP (52 kD) were expressed at 20, 30 and 37°C using the same backbone vector (pETM vectors [18]) and BL21 (DE3) co-transformed with the β-galactosidase probe. The first three proteins are very soluble at any of these temperatures, whilst the GFP fusion aggregates when the growth temperature is above 30°C [16]. A good correlation was observed between β-galactosidase activities and the intensity of the bands stained in SDS-gels loaded with the samples corresponding to soluble fractions and pellets (data not shown). In particular the data indicated that the activation of the β-galactosidase probe specifically correlates with protein aggregation and is not influenced by the protein mass. NusA and the GST-GFP fusion have a similar mass but only cells transformed with GST-GFP and grown at 37°C showed a significant increase of the β-galactosidase activity.

Next, two model proteins were selected to test the probe at different ranges of aggregation. ClipA5 was chosen as insoluble protein beside the soluble GST and both proteins were cloned in an array of vectors differing for the features that control the rate of expression and its regulation (Table 1). Cells pelleted during their exponential growth phase were used to measure β-galactosidase activity.

**Table 1:** List of the constructs used in the experiments. The features of the vectors are indicated along with the expressed insert.

Vector	Insert	Promoter	Inducer	Origin
pBADM	GST, ClipA5	*ara*BAD	arabinose	pUC
pTrcHis2	GST, ClipA5	*trc*	IPTG	pUC
pKK223	GST, ClipA5	*tac*	IPTG	pBR322
pQE30	GST, ClipA5	T5/*lac*	IPTG	colE1
pETM	GST, ClipA5	T7/*lac*	IPTG	pBR322
pGAT	GST	T7	IPTG	pUC
pZS24MCS	GST, ClipA5	P*lac*/*ara-1*	arabinose	pSC101

The measurements were highly reproducible among independent purifications and independent of the used constructs (Fig. 1B). As expected, the expression of the aggregation-prone ClipA5 correlated with a higher β-galactosidase activity when the cells were grown at 30 and 37°C, whilst 90 min of expression at 20°C were not sufficient to induce a significant accumulation of ClipA5 aggregates. In contrast, GST-dependent β-galactosidase activity was detected only when the bacteria were grown at 37°C. The choice of the vector influenced the level of aggregation, but not the reproducibility of the data collected using the same material.

In these experiments the maximal differences among independent measurements of the same expression combination were less than 12% at 37°C and 5% at lower temperatures, making them statistically negligible.

### Range of reliable application of the β-galactosidase probe

β-galactosidase assay appeared, therefore, reliable over an array of experimental conditions and further aspects of its possible applications were tested.

**Figure 2:**
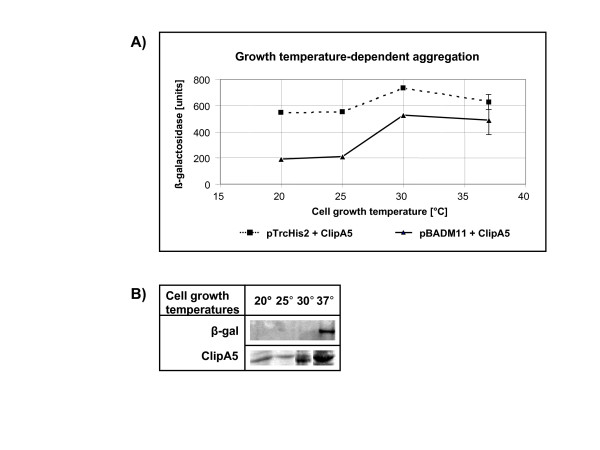
Conditions limiting the use of the β-galactosidase probe. A) ClipA5 aggregation estimated by β-galactosidase activity following protein expression from pBADM11 and pTrcHis2 vectors and cells incubated at different temperatures. The results are the average of three independent measurements. B) The accumulation of ClipA5 in the pellet recovered from bacteria co-transformed with pTrcHis2 and *Ibp*AB-β-galactosidase and cultured at different temperatures was identified by SDS-PAGE, whilst β-galactosidase precipitates were detected by western blot.

Leaky expression of recombinant proteins in the absence of inducer may be deleterious in some cases, for instance when toxic proteins must be produced [19]. The *ara*BAD promoter is considered better controlled than the *trc *but, since leakage is generally very limited, its accurate measurement by the β-galactosidase assay would further confirm the sensitivity of the method. The aggregation of ClipA5 expressed from pTrcHis2 and pBADM11 was compared at different temperatures. At 20 and 25°C the induction-dependent accumulation of the recombinant protein was as low as it was undetectable after SDS-PAGE (data not shown). Consequently, the difference of β-galactosidase activity is mostly to attribute to the leakage of the *trc*-based system (Fig. 2A). The aggregation increased in both systems when the bacteria were grown at 30°C, and the absolute difference was reduced. This was probably due to the contribution of the induced expression at this temperature which partially covered the leakage background.

Surprisingly, the β-galactosidase activity decreased when the bacteria were grown at 37°C, namely at conditions expected being more favorable for larger aggregation, and the reproducibility of the β-galactosidase activity assay became significantly lower than in average. These unexpected results urged to verify the reliability of the assay under these experimental conditions using an alternative method. First, the ClipA5 content in the insoluble fractions from the different cultures was directly analyzed by SDS-PAGE. ClipA5 aggregates showed a temperature-dependent increase (Fig. 2B), thus confirming the indications of the β-galactosidase assay performed with bacteria cultured at 20 and 30°C, but contradicting the apparent decrease measured by β-galactosidase activity in bacteria grown at 37°C. Therefore, the hypothesis of a partial precipitation of the aggregation reporter was tested by Western Blot. It turned out that some β-galactosidase co-precipitated when the bacteria expressing the insoluble ClipA5 were cultured at 37°C (Fig. 2B).

Considering together the data of Figures 1B and 2A, the temperature of 37°C seems to be non-permissive only in combination with elevated aggregation (more than 600–800 activity units). Nevertheless, we decided to run the successive experiments at 30°C because no drawbacks were identified at such a temperature.

Lesley and coworkers always cultured their bacteria at 37°C and the fact that β-galactosidase can co-precipitate in the presence of elevated aggregation could explain the underestimation of insoluble constructs reported in their work [17].

### Evaluation of the expression vectors allowed by the sensitivity of the probe

The vectors listed in Table 1 cover almost completely the range of the features used to build conventional expression plasmids and some of them, like origin of plasmid replication or kind of promoter, strongly contribute to the expression rate of the recombinant protein and, consequently, can influence the folding efficiency.

**Figure 3:**
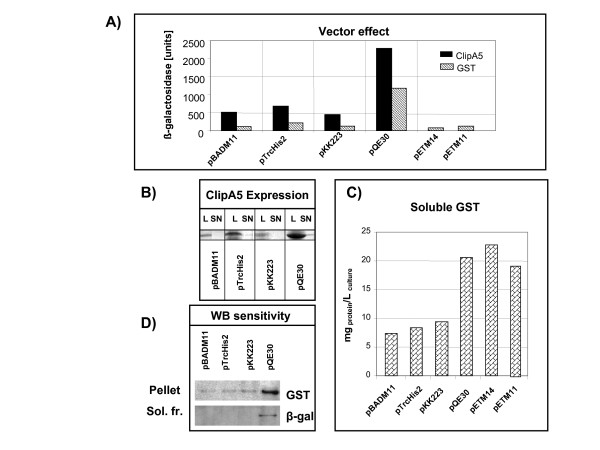
Effect of the vector features on the aggregation of the expressed protein. A) β-galactosidase-estimated levels of ClipA5 and GST aggregates accumulated after expression of the proteins at 30°C using different vectors. The reported results are the average of 3 independent experiments with SD lower than 5%. B) ClipA5 detection by SDS-PAGE using total lysate (L) and soluble supernatant (SN) from cells transformed with different expression vectors. C) Yields of soluble GST expressed by different vectors. D) Western blots to identify the precipitated GST and the soluble β-galactosidase in cultures grown at 30°C and transformed with different vectors expressing GST.

The choice of the right vectors for cloning a specific protein goes behind the aims of this work. However, from a practical point of view it is of much interest to identify which of their parameters can control the protein solubility. Densitometry after SDS-PAGE can be used to quantify the proteins in the different fractions. However, in our hands, the estimation of ClipA5 aggregate by densitometry measurements raised a high variability at both saturating and very low protein concentrations. The differences of β-galactosidase activity among the replicates of a single experimental condition reported in Figure 3A were in the range of 5%, but an operator-dependent variability of 20–30% was measured when densitometry was used with the samples of Figure 3B.

In contrast, the first experiments reported in this work demonstrated that the β-galactosidase probe is sensitive enough to distinguish among minor differences in protein aggregation and gives very reproducible data. Therefore, the two model proteins were cloned into a set of different vectors and co-transformed with the *Ibp*AB-promoter β-galactosidase fusion for efficiently evaluating and comparing the levels of aggregation.

The cells were grown at 30°C and the results summarized in Figure 3A show that the insoluble ClipA5 always induced higher β-galactosidase accumulation than the more soluble GST, but that there were striking differences when the same protein was expressed using different vectors.

This is particularly true for the GST expressed in the very similar pETM vectors 11 and 14 (Fig. 3A). This example is particularly interesting because it shows the accuracy of the method even when the measured absolute differences are low. In a previous work it was shown that pETM11 produced less soluble protein than pETM14 and this result was correlated to the longer and more hydrophobic linker connecting the tag to the target protein [18]. The aggregation data estimated by the β-galactosidase assay confirm now that GST forms a double amount of aggregates when expressed from pETM11 rather than from pETM14.

However, no direct correlation was found for any other single vector feature and protein insolubility (Table 1 and Fig. 3A). For instance, the plasmid copy number is a function of the origin of replication and pUC, present in pBADM11 and pTrcHis, determines the highest plasmid concentration in the cell. The elevated number of plasmids is a parameter that can increase the expression rate and, consequently, the protein aggregation but, in combination with *ara*BAD and *trc *promoters, the resulting proteins aggregated less than when expressed by the lower represented plasmid pQE30 (colE1) coupled to T5/lac promoter.

A large-scale purification was performed in parallel to exactly calculate the yields of the soluble GST produced using the different vectors (Fig. 3C) and to verify the expected inverse correlation between estimated aggregation and measured amounts of soluble protein. In the case of pETM14 the highest yield of soluble protein perfectly correlated with the lowest aggregation measured by the β-galactosidase probe. Such a correlation was confirmed for most of the other vectors that produced comparable amounts of total recombinant protein. An exception was represented by pQE30, namely the vector that enabled the highest rate of recombinant expression and that yielded large amounts of both soluble and aggregated GST.

Therefore, all together the collected data suggest that, even though no single vector feature alone is responsible for the solubility of the expressed proteins, the total aggregation tends to increase when the complex of the elements involved in the expression regulation allows a higher expression rate. However, elevated aggregation does not automatically mean low level of total soluble protein.

A specific feature of the β-galactosidase test performed in our experiments is that it enables the identification of aggregates in a very early stage during cell growth. Therefore, this analysis speeds up the screening for improved solubility at a moment in which the low bacterial density together with the short expression time result in protein concentrations difficult to detect by SDS-PAGE and even by western blot. For instance, the insoluble GST accumulated in the pellet of the bacteria transformed with pQE30 was clearly identified in a western blot, but only a faint shadow was observed when samples from bacteria that accumulated less aggregates were loaded (Fig. 3D) and the signal of β-galactosidase in the soluble fraction was almost undetectable even using the supernatant from the most aggregating fractions (Fig. 3D).

### Optimization of the expression conditions using the β-galactosidase

Even though the data indicating total yields of soluble protein cannot be directly inferred by the amount of aggregates, the recombinant expression protocols can be improved observing the variation in aggregation in response to specific factors, like growth temperature. Therefore, further experiments were conceived to evaluate the correlation between variations of the aggregation degree and yields of soluble proteins inside the same expression system.

**Figure 4:**
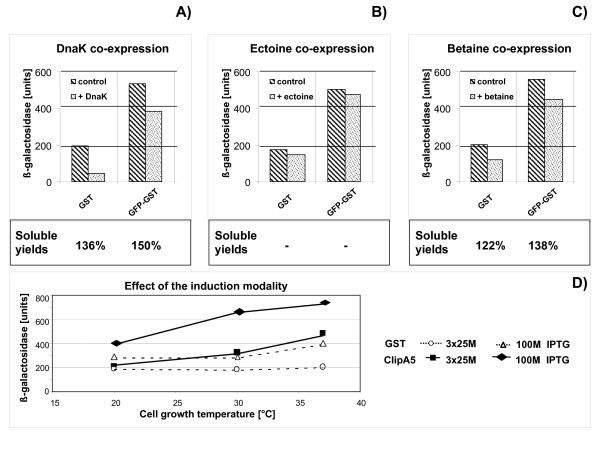
Effect of molecular and chemical chaperones on the solubility of recombinantly expressed proteins. GST was expressed using the pKK223 vector, GST-GFP by a gateway vector and the effect of DnaK (A) co-expression, 10 mM ectoine (B) and 5 mM betaine + 0.4 M NaCl (C) addition was measured. Soluble proteins were purified, the yields compared between control and treated samples and the variations induced by the growth condition modifications were calculated (control = 100%). D) β-galactosidase activity induced by the expression of GST and ClipA5 cloned into the pTrcHis2 vector and induced at 3 different temperatures either once with 100 μM IPTG or 3 successive times with 25 μM IPTG. All the experiments were repeated at least three times (SD < 4%).

In a previous work we showed that the over-expression of DnaK reduced the mass of the GST-GFP aggregates [16]. Now the aggregation of both GST and GST-GFP constructs was indirectly measured using the β-galactosidase assay and the stabilizing effect of DnaK co-expression was confirmed (Fig. 4A). In parallel, the soluble protein was purified from the different bacteria cultures and estimated lower aggregation always correlated with higher yields of soluble protein. Next we added the osmolytes ectoine and betaine to the culture medium for improving the solubility of the recombinantly expressed proteins [20,21]. Only the treatment with betaine strongly decreased the aggregation and resulted in a higher amount of soluble protein (Figs. 4B and 4C). It is interesting that in all the three cases the evaluation of the aggregate performed by β-galactosidase assay in the early stage of recombinant expression strongly correlated with the final yield results.

The rate of recombinant expression is dependent on the inducer concentration and, at high expression rates, the folding capacity of the cell can become limiting, with an increased amount of protein that can misfold and aggregate. Two inducing strategies have been compared: in the first case IPTG was added to the cell culture three times using non-saturating concentrations (25 μM); in the second a single addition of 100 μM IPTG was used. The first strategy resulted more effective in preventing the aggregation of both proteins, even though the aggregation-sensitive ClipA5 always triggered a higher β-galactosidase activity than GST (Fig. 4D). The differences remained limited but 14% more soluble GST was recovered when IPTG was fractionated (data not shown).

## Conclusion

The collected data considerably widen the range of application of the β-galactosidase probe [17] as a reliable reporter to estimate the degree of aggregation of recombinant proteins expressed in bacteria. The system resulted more sensitive and faster than a SDS-PAGE followed by western blot analysis or densitometry, suitable to detect the aggregation in the early stage of the cell culture, and reliable to identify even minor variations of the aggregate amount. Furthermore, it enabled the establishment of a correlation between the aggregation decrease due to cell culture optimization and the improved yields of soluble proteins. It also showed that the absolute level of recombinant expression rather than single features of the expression vector is responsible for high aggregation. This observation is of practical interest because it could lead to misuse of the results obtained with the β-galactosidase probe. It would be the case of constructs producing high amounts of both soluble and aggregated protein (pQE in Fig. 3A and 3C) that would be excluded after a solubility screening based only on the estimated aggregation. In conclusion, the co-expression of the β-galactosidase probe together with a protein of interest can simplify the identification of the optimal expression conditions, but the limits of its application must be known and this work contributed to identify them.

Notably, we showed that the probe itself can precipitate in the presence of large aggregates leading to underestimation of the aggregation degree. This fact could explain the 20% of inconsistent results mentioned in the original paper [17]. Indeed, the reproducibility of the data was always higher when the bacteria growth temperature was in the range of 20–30°C rather than 37°C, an aggregation-prone condition.

Probes for the aggregation detection have been proposed in the past [11-14], but most of them were fused to the protein of interest and a direct interaction effect could not be ruled out. A non invasive probe was built by fusing the promoter element of the heat shock transcription factor σ^32 ^with the GFP and used to estimate the cell response to stress [22]. However, GFP needs 95 min to fold and, because of such a delay between trigger signal and its possible identification, accurate measurements were limited to resting cells when no mass variations have to be taken in account. In contrast, the β-galactosidase probe is particularly useful to identify aggregation since the early growth stage and, because of its specificity for aggregates, allows to distinguish when low yields of soluble recombinant proteins are due to protein precipitation or to other reasons, like cell negative selection, mRNA instability or protein degradation.

Finally, this aggregate detection system can be used for studying theoretical problems, like relationship between cell stress and protein aggregation. Because of its sensitivity even in the early stage of aggregate development a probe with analogous features can be envisaged as a reporter in eukaryotic systems to monitor the presence of disease-related primordial aggregates.

## Methods

### Cloning, cell culture and protein analysis

Thioredoxin, NusA, and GST were directly expressed using the pETM vectors 22, 66, and 33 [18]. The GST-GFP fusion construct was described in [23] and the pGAT used to produce GST in [24]. The serine protease ClipA5 (EAA00427) and the GST were cloned between NcoI and EcoRI in the vectors pBADM11, pTrcHis2 (both Invitrogen), pETM 11 and pETM14 (both [18]), between Acc65I and MluI in pZS24MCS1 [25], between Acc65I and HindIII in pQE30, and EcoRI and HindIII in pKK223 (GE Healthcare).

Positive clones were used to transform Top10 (pBADM and p ZS24MCS1), XL-Blue (pTrcHis2, pKK223, and pQE30) or BL21(DE3) (pETM and pGAT) cells and the expression was preliminary verified in bacteria cultured at 37°C in the presence of kanamycin (constructs cloned in pETM and pZS24MCS1) or carbenicillin (all the others) and induced 2 hours with 0.5 mM IPTG. The plasmid pHK57 containing the *ibp*B-promoter β-galactosidase fusion [17] was co-transformed in positively tested bacteria. Glycerol stocks (20%) were frozen and used to inoculate over-night culture cultured at 30°C in the presence of 1% glucose. The pre-culture was added 1:100 to flasks containing 10 mL of Lauria Bertani medium that were initially incubated at 37°C in an orbital shaker. The temperature was lowered to 30, 25 or 20°C at a bacterial OD_600 _= 0.2 and the recombinant expression was induced by arabinose (1,5 mg/mL) or IPTG (0.1 mM) after further 45 min. The cells were pelleted 90 min after induction. In the case of multiple induction with 25 μM, the second and third induction were performed 30 min and 60 min after the first treatment. DnaK was co-expressed using the pBB550 vector, whilst ectoine (final concentration 10 mM) and betaine (5 mM plus 0.4 M NaCl) were directly added to the culture media [21].

Bacteria aliquots corresponding to 2 mL of culture were recovered, lysated and the recombinant protein affinity purified as described in [18]. Total lysates and pellets were separated by SDS-PAGE and, when necessary, blotted onto a nitrocellulose membrane. GST was detected using a mouse anti-GST primary antibody and a goat anti-mouse HRP conjugated secondary antibody (Sigma). The His-tag present in both the GST and ClipA5 constructs was directly identified using anti-His, HRP conjugated antibodies (Sigma). The β-galactosidase was detected using mouse anti β-galactosidase primary antibodies (Qiagen) and the goat anti-mouse HRP conjugated as secondary antibodies (Sigma). The development of the signal was obtained using ECL Western Blotting reagents (GE Healthcare).

Large scale purification of GST was performed starting from the pellet corresponding to 1 L growth medium cultured overnight at 20°C and the protein was purified by glutathione affinity chromatography using a HiTrapGST column and a FPLC equipment (GE Healthcare).

### β-galactosidase activity

The protocol follows the method proposed by Miller [26]. Frozen pellets were resuspended in 2 mL PBS buffer and the absorbance at 600 nm was used to detect the cell density. 500 μL of cell suspension was mixed 1:1 with 50 mM Na-phosphate buffer, pH 7.0, 10 mM KCl, 1 mM MgSO_4_, 50 mM β-mercaptoethanol before addition of 3 μL DMSO and 3 μL 10% Na-deoxycholate. The suspension was vortexed, incubated 10 min at 37°C and the resulting lysate 5 min at 28°C. The reaction was started by the addition of 200 μL of ortho-nitrophenyl-β-galactoside (4 mg/mL). After vortexing the suspension turned yellow and was stopped by the addition of 500 μL of 1 M Na_2_CO_3_. The OD_420_, the OD_550 _and the reaction time were recorded and used along with the initial OD_600 _value to calculate the β-galactosidase activity expressed in units:

1000 × (OD_420 _– 1.75 × OD_550_)/t × culture volume × OD_600_

The background values (β-galactosidase activity of cells not harboring an expression plasmid) were subtracted to obtain the specific activity data.

## Competing interests

The authors have no competing interests.

## Authors' contributions

LM cloned most of the constructs, set the enzymatic assay and performed the preliminary measurements. TS completed the cloning and carried out most of the experiments. AdM designed the work, completed the experiments, and wrote the manuscript. All the authors read and approved the final manuscript.
